# Supplementary material for: Unusually high thermal conductivity in suspended monolayer MoSi2N4
Source: Nat Commun. 2024 Jun 6;15:4832. doi: 10.1038/s41467-024-48888-9 (PMC11156898; doi:10.1038/s41467-024-48888-9)
Supplement: Supplementary file 1 — Supplementary Information [file 41467_2024_48888_MOESM1_ESM.pdf]

Supplementary information for

Unusually high thermal conductivity in suspended monolayer $MoSi_2N_4$

Chengjian He[1,2]†, Chuan Xu[1,2]†, Chen Chen[1,2]†, Jinmeng Tong[1,2], Tianya Zhou[1,2], Su Sun[1,2], Zhibo Liu[1,2], Hui-Ming Cheng[1,2,3], Wencai Ren[1,2]*

[1] Shenyang National Laboratory for Materials Science, Institute of Metal Research, Chinese Academy of Sciences, Shenyang 110016, P. R. China.

[2] School of Materials Science and Engineering, University of Science and Technology of China, Shenyang 110016, P. R. China.

[3] Shenzhen Institute of Advanced Technology, Chinese Academy of Sciences, Shenzhen 518055, China.

† These authors contribute equally: Chengjian He, Chuan Xu, Chen Chen

* Corresponding author. E-mail: wcren@imr.ac.cn

The supplementary information includes:

Supplementary Figures 1-17

Supplementary Tables 1-5

Supplementary Notes 1-3

Supplementary References

Supplementary Figures

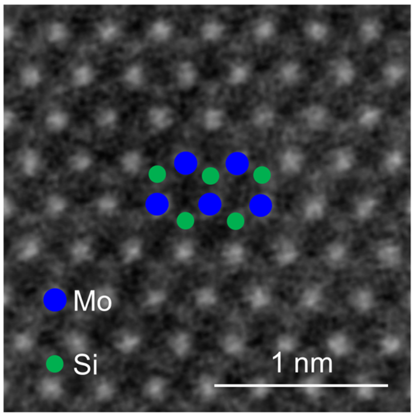

Supplementary Fig. 1 | In-plane atomic structure of monolayer $MoSi_2N_4$. Atomic level resolution HAADF-STEM image of monolayer $MoSi_2N_4$ crystal, showing the atomic sites of Mo (blue balls) and Si (green balls).

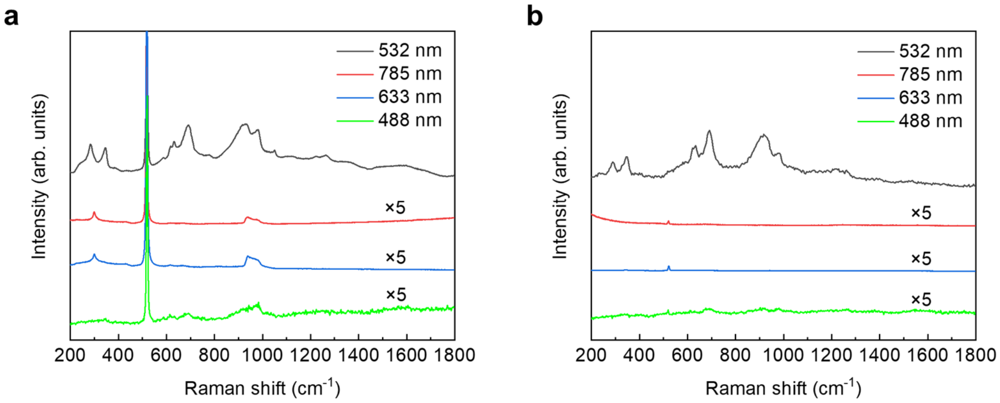

Supplementary Fig. 2 | Raman spectra of monolayer $MoSi_2N_4$ supported on SiO_2/Si substrate (**a**) and suspended on through holes (**b**) under different excitation wavelengths of 532 nm, 785 nm, 633 nm and 488 nm.

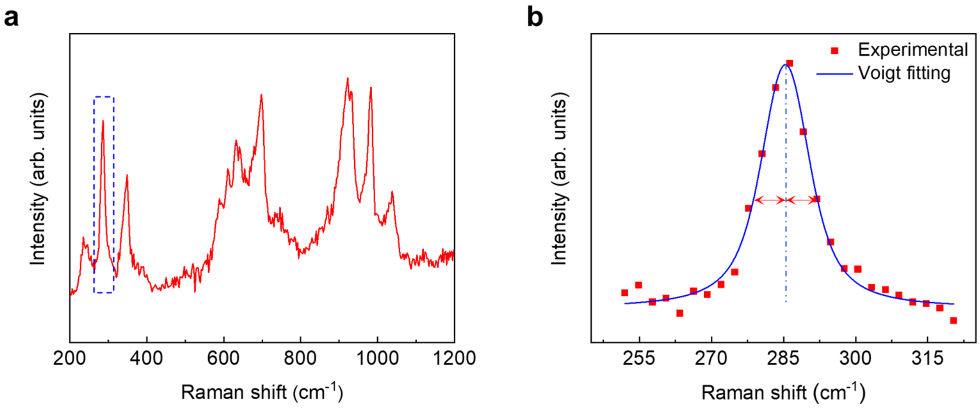

Supplementary Fig. 3 | Precise identification of the Raman peak position. a, Raman spectrum of a monolayer $MoSi_2N_4$ flake suspended on holey substrate under 532 nm incident laser. **b,** SN_1 mode extracted from **a** with a Voigt function fitting.

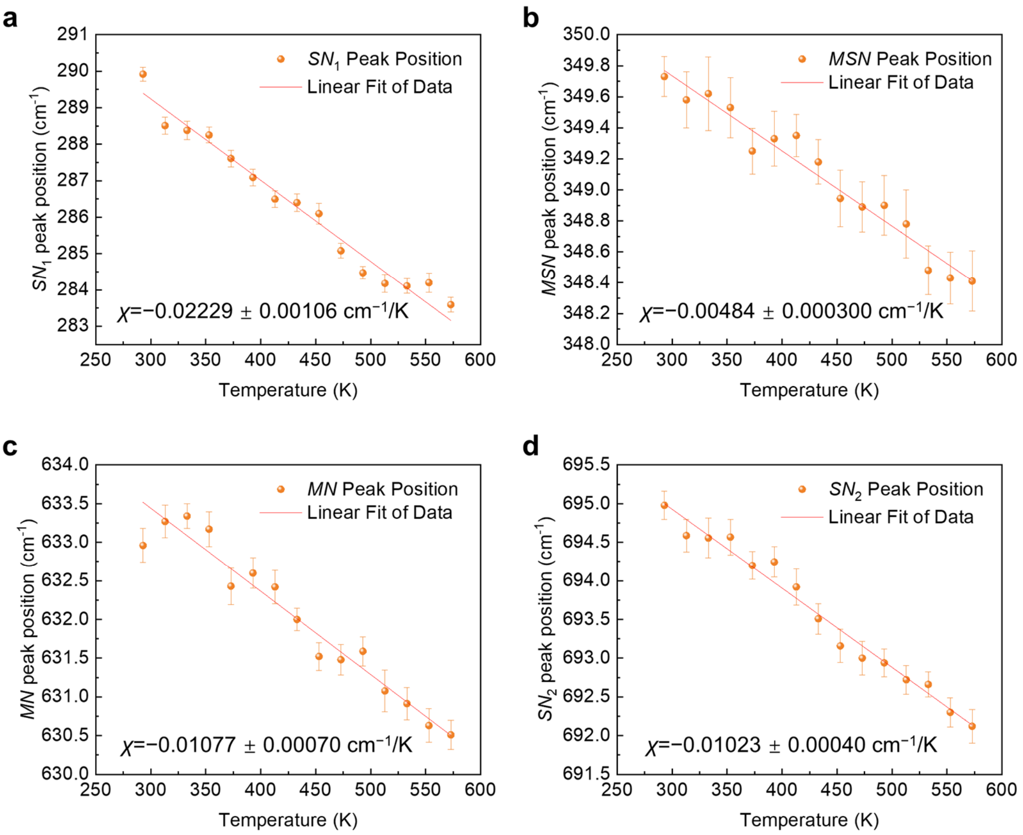

Supplementary Fig. 4 | Linear fittings of temperature dependent frequency of Raman SN_1 (a), *MSN* (b), *MN* (c) and SN_2 (d) mode of $MoSi_2N_4$ on SiO_2/Si, respectively. Error bars in **a-d** represent standard deviations from Voigt function fitting in identifying the accurate Raman peak positions.

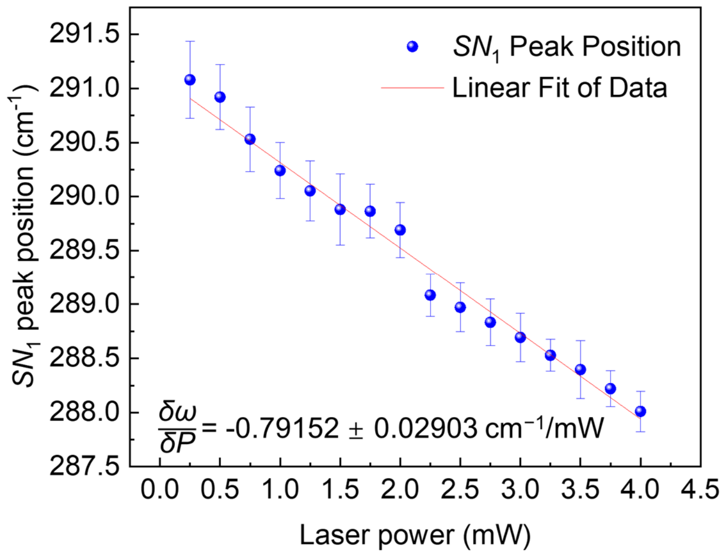

Supplementary Fig. 5 | Linear fitting of laser power dependent frequency of Raman SN_1 mode of monolayer $MoSi_2N_4$ on SiO_2/Si. Error bars represent standard deviations from Voigt function fitting in identifying the accurate Raman peak positions.

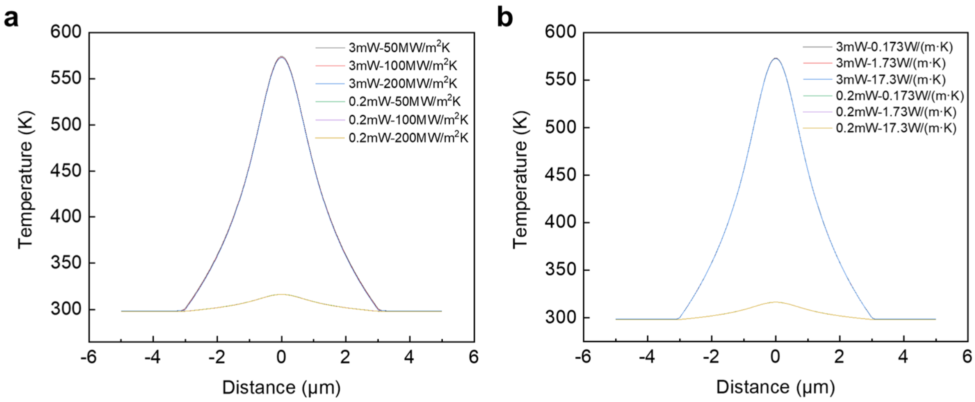

Supplementary Fig. 6 | Temperature profiles across the monolayer $MoSi_2N_4$ at different excitation laser power, interface thermal conductance and out-of-plane thermal conductivity under the hole diameter of 6 μm. a, With a constant out-of-plane thermal conductivity of 0.173 $W·m^{-1}·K^{-1}$; **b,** With a constant interface thermal conductance of 100 $MW·m^{-2}·K^{-1}$.

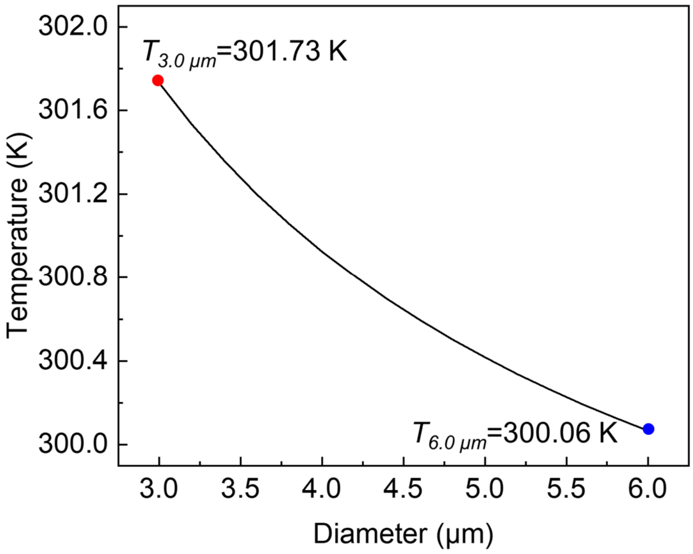

Supplementary Fig. 7 | Hole-diameter dependent temperature of suspended monolayer $MoSi_2N_4$ near the hole edge. As the hole diameter increases, the temperature of monolayer $MoSi_2N_4$ near the hole edge decreases gradually to ambient temperature.

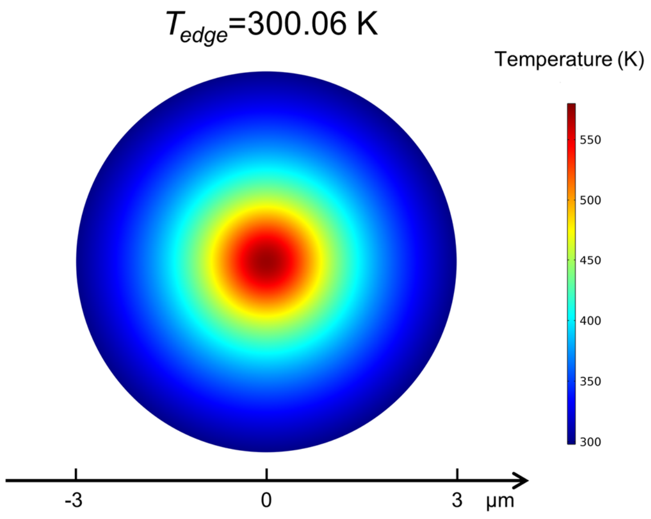

Supplementary Fig. 8 | Simulated spatial temperature distribution of monolayer $MoSi_2N_4$ suspended on a 6-μm-diametered hole at an excitation power of 3 mW.

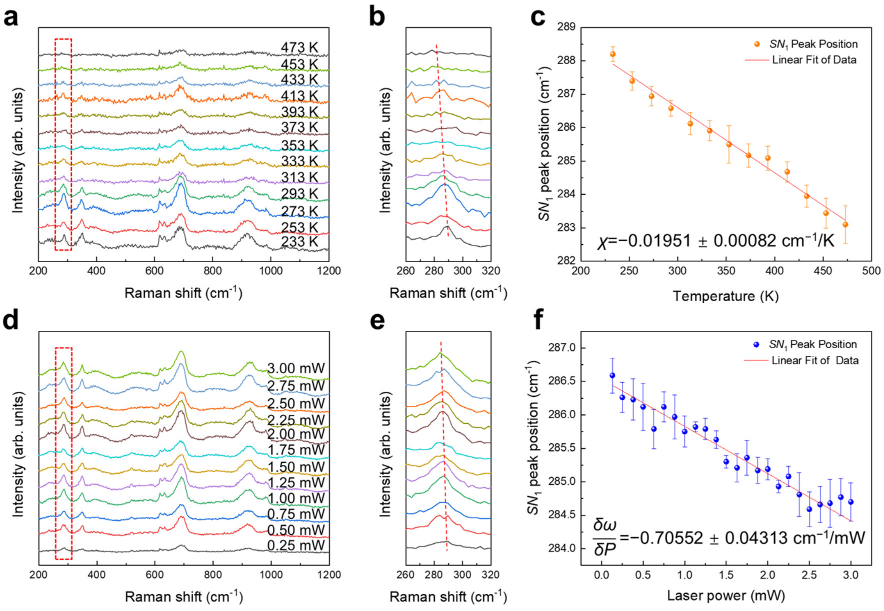

Supplementary Fig. 9 | Temperature- and laser power- dependent Raman spectra of suspended monolayer $MoSi_2N_4$ measured in nitrogen gas environment. a, d, Raman spectra evolutions with temperature (**a**) and incident laser power (**d**), respectively, where SN_1 modes were marked by red dotted box. **b, e,** The corresponding SN_1 mode evolution with temperature (**b**) and incident laser power (**e**), respectively. **c,f,** The linear fittings of SN_1 mode shifts as a function of temperature (**c**) and incident laser power (**f**), respectively. The red dotted lines in **b** and **e** represent the shift trend of Raman modes. Error bars in (**c**) and (**f**) represent standard deviations from Voigt function fitting in identifying the accurate Raman peak positions.

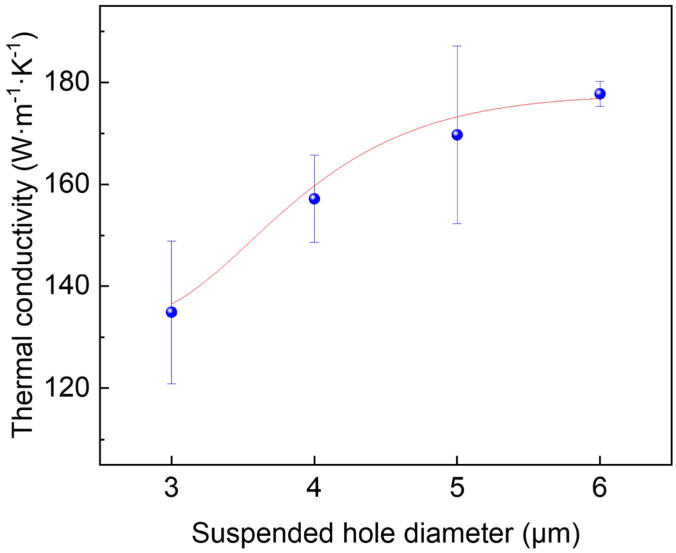

Supplementary Fig. 10 | The measured thermal conductivities of monolayer $MoSi_2N_4$ suspended on holes with the diameter of 3, 4, 5, and 6 µm. The red curve represents a log function fitting. Error bars represent standard deviations of thermal conductivity extraction from optothermal Raman measurements.

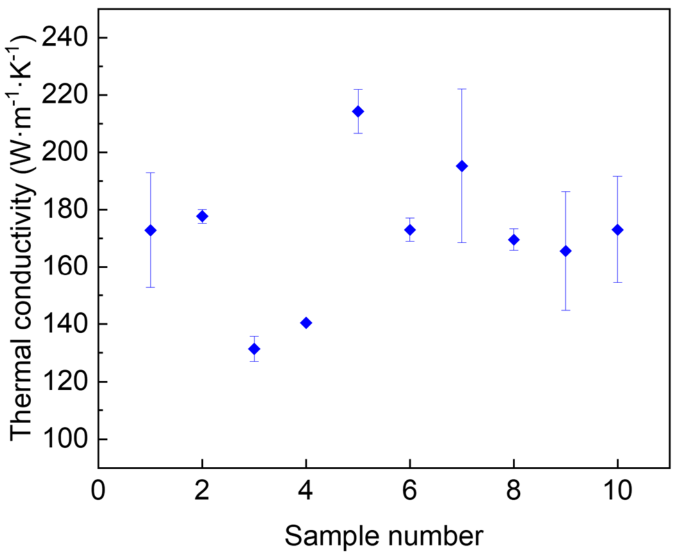

Supplementary Fig. 11 | The measured thermal conductivities of 10 suspended monolayer $MoSi_2N_4$ samples from different batches. Error bars represent standard deviations of thermal conductivity extraction from optothermal Raman measurements.

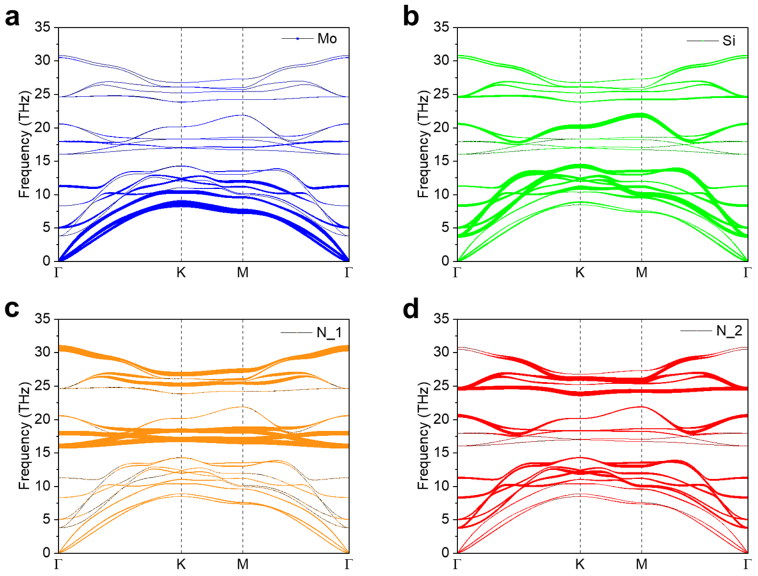

Supplementary Fig. 12 | Atomic projected phonon dispersions of monolayer $MoSi_2N_4$. a, Mo; **b,** Si; **c,** N_1; **d,** N_2.

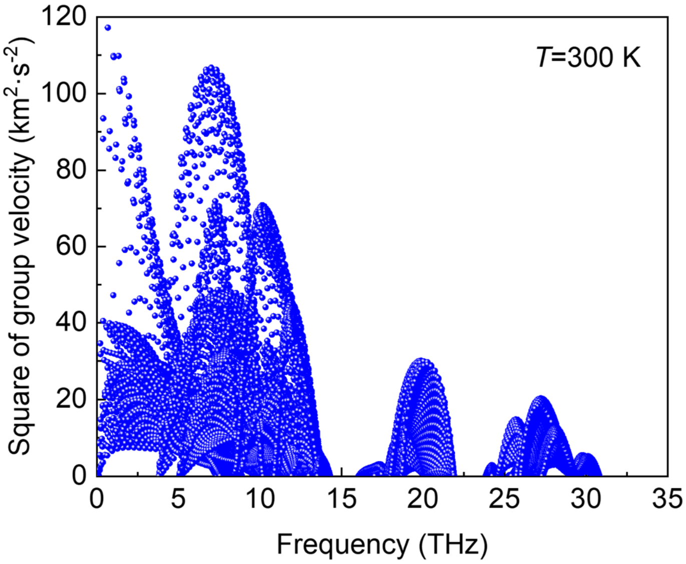

Supplementary Fig. 13 | Frequency-dependent square of group velocities of monolayer $MoSi_2N_4$ at 300 K.

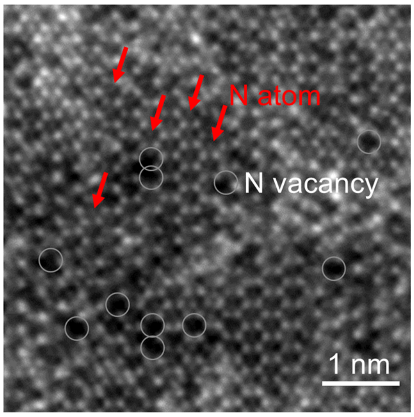

Supplementary Fig. 14 | In-plane atomic-level iDPC-STEM image of monolayer $MoSi_2N_4$. The red arrows indicate the nitrogen atoms in the center of the honeycomb lattice, while the white circles denote the nitrogen vacancies.

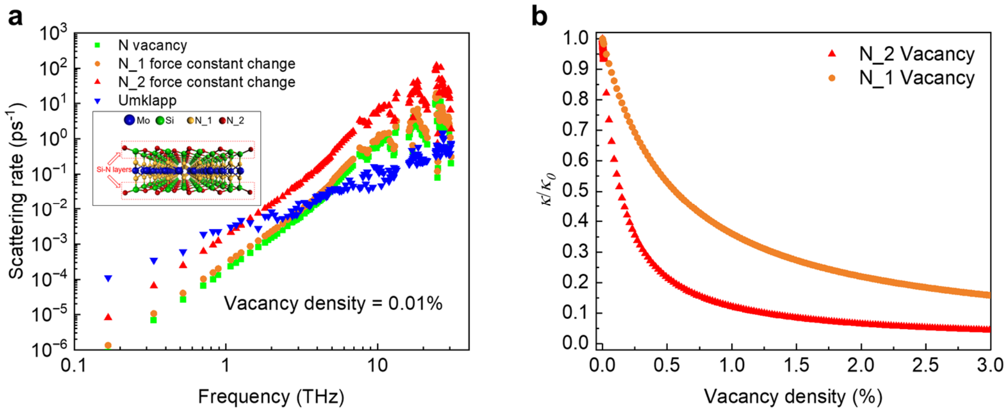

Supplementary Fig. 15 | Phonon scattering rate and thermal conductivity of defective $MoSi_2N_4$ at 300 K. a, The phonon scattering rate as a function of phonon frequency with a N vacancy density of 0.01%. The inset shows the side view of the atomic structure of monolayer $MoSi_2N_4$. **b,** The ratio of the thermal conductivity of defective $MoSi_2N_4$ to that of pristine one at room temperature as a function of N vacancy density.

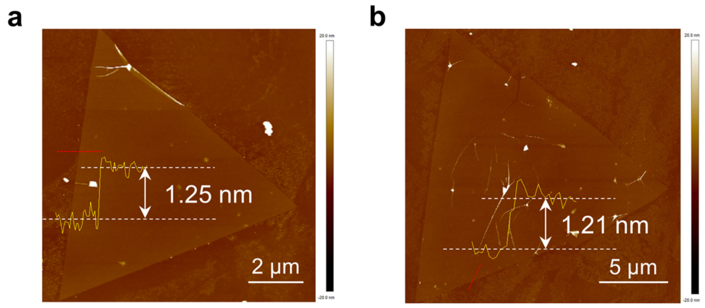

Supplementary Fig. 16 | AFM images of monolayer $MoSi_2N_4$ single crystals on SiO_2/Si. a, A small flake with a lateral size of ~9.3 μm. **b,** A large flake with a lateral size of ~20.2 μm. More wrinkles can be observed on the larger flake (**b**) than the smaller one (**a**).

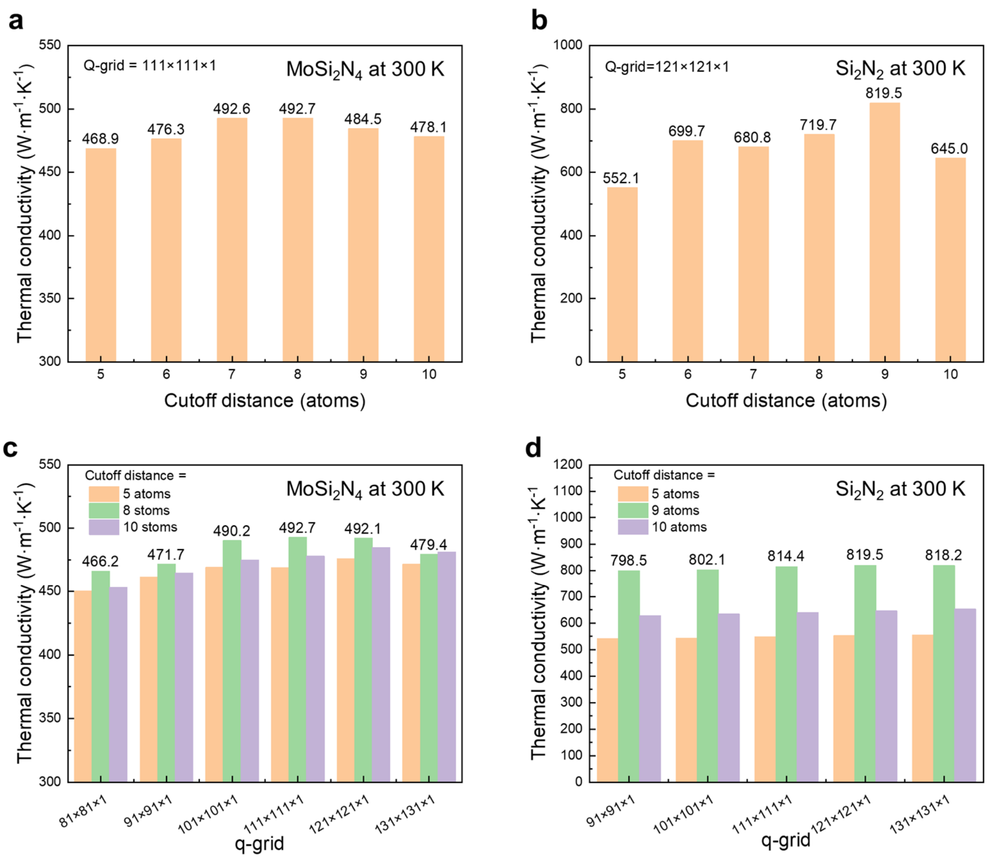

Supplementary Fig. 17 | The convergence tests of cutoff distance and q-grid of $MoSi_2N_4$ and Si_2N_2 at 300 K. a,b, The convergence tests of cutoff distance of $MoSi_2N_4$ with a q-grid of 111 × 111 × 1 (**a**) and Si_2N_2 with a q-grid of 121 × 121 × 1 (**b**). **c,d,** The convergence tests of q-grid of $MoSi_2N_4$ with cutoff distances of 5, 8, 10 atoms (**c**) and Si_2N_2 with cutoff distances of 5, 9, 10 atoms (**d**).

Supplementary Tables

Supplementary Table 1 | Comparison of thermal conductivities of monolayer $MoSi_2N_4$ with other 2D semiconductors.

Materials	Atoms per unit cell	Average atomic mass	Thermal conductivity ($W \cdot m^{-1} \cdot K^{-1}$)	References
Bi_2O_2Se	5	105.8	0.92	*(1)*
			1.6	*(2)*
$FePS_3$	5	36.6	1.35	*(3)*
Ta_2NiS_5	8	72.6	z: 4.76<br>a: 7.79	*(4)*
$TaSe_2$	3	113.0	9	*(5)*
InSe	4	96.9	28.7	*(6)*
WS_2	3	82.7	32	*(7)*
MoS_2	3	53.3	32.5	*(8)*
			40.8	*(9)*
			84	*(10)*
$PtSe_2$	3	117.7	40.4	*(11)*
WSe_2	3	113.9	43	*(12)*
$MoSe_2$	3	84.7	59	*(10)*
PtS_2	3	86.3	85.6	*(11)*

Black phosphorus	4	31.0	z: ~20<br>a: ~10	*(13)*
Ti_2CO_2	5	27.9	40.58*	*(14)*
$MoSi_2N_4$	7	29.7	173.03	This work

Notes:

z: Zigzag-edge direction; a: Armchair direction.

The thermal conductivity of Ti_2CO_2 was derived from theoretical calculation, while those of other materials were measured based on the optothermal Raman technique.

Supplementary Table 2 | The $\chi/(\delta\omega/\delta p)$ and the derived thermal conductivities of monolayer $MoSi_2N_4$ suspended on holes with the diameter of 3, 4, 5 and 6 μm.

Hole diameter (μm)	$\chi/(\delta\omega/\delta p)$ ($mW\cdot K^{-1}$)	Thermal conductivity ($W\cdot m^{-1}\cdot K^{-1}$)
3	0.016426 ± 0.001706	134.87 ± 14.00
4	0.019141 ± 0.001042	157.16 ± 8.55
5	0.020670 ± 0.002118	169.71 ± 17.39
6	0.021649 ±0.000295	177.75 ± 2.42

Supplementary Table 3 | The χ /($\delta\omega/\delta p$) and the corresponding thermal conductivity values of 10 suspended monolayer $MoSi_2N_4$ samples.

Sample number	χ /($\delta\omega/\delta p$) ($mW\cdot K^{-1}$)	Thermal conductivity ($W\cdot m^{-1}\cdot K^{-1}$)
1	0.021048 ± 0.002448	172.81 ± 20.10
2	0.021649 ± 0.000295	177.75 ± 2.42
3	0.016000 ± 0.000524	131.37 ± 4.30
4	0.017109 ±0.000025	140.47 ± 0.21
5	0.026097 ± 0.000937	214.28 ± 7.69
6	0.021074 ± 0.000492	173.03 ± 4.04
7	0.023785 ± 0.003257	195.29 ± 26.74
8	0.020653 ± 0.000454	169.58 ± 3.73
9	0.020174 ± 0.002525	165.64 ± 20.73
10	0.021077 ± 0.002269	173.05 ± 18.63

Supplementary Table 4 | Comparison of Young's modulus of monolayer $MoSi_2N_4$ with other 2D semiconductors shown in Supplementary Table 1.

**Materials**	**Method**	**Young's modulus (GPa)**	**References**
Bi_2O_2Se	AFM	~65	*(15)*
InSe	AFM	101.37±17.93	*(16)*
$MoSe_2$	AFM	177.2 ± 9.3	*(17)*
WSe_2	AFM	258.6±38.3	*(18)*
MoS_2	AFM	270 ±100	*(19)*
WS_2	AFM	272 ± 18	*(20)*
$FePS_3$	DFT	51	*(21)*
$TaSe_2$	AFEM	a: 120.3 (80.7 $N·m^{-1}$)<br>z: 123.84 (83.1 $N·m^{-1}$)	*(22)*
PtS_2	MD	178.1 (99.7 $N·m^{-1}$)	*(23)*
Ti_2CO_2	DFT	~545	*(24)*
Black phosphorus	First-principles calculations	a: 41.3<br>z: 106.4	*(25)*
$MoSi_2N_4$	AFM	491.4 ± 139.1	*(26)*

Notes:

AFEM: Atomic finite element method; MD: Molecular dynamics simulation;

a: Armchair direction; z: Zigzag-edge direction;

The original calculation results of $TaSe_2$ and PtS_2 were shown as 2D elastic modulus

(N·m^{-1}). For comparison, we took into account their thicknesses (https://materialsproject.org) and converted 2D elastic modulus to Young's modulus (GPa).

Supplementary Table 5 | Comparison of scaling parameter of $MoSi_2N_4$ with other 2D semiconductors shown in Supplementary Table 1.

Materials	$\bar{M}$	δ (10^{-10}m)	θ (K)	γ	$\bar{M}\delta\theta^3$ (m·K^3)	θ^3/γ^2 (10^6 K^3)	κ (W·m^{-1}·K^{-1})	*References*		
								θ	γ	κ
MoS_2	53.3	2.70	262.3	~1.22	0.25969	12.12482	84	*(27)*	*(27)*	*(10)*
$MoSe_2$	84.7	2.78	177.6	~1.20	0.13209	3.89015	59	*(27)*	*(27)*	*(10)*
WS_2	82.7	2.67	213.6	~1.15	0.21504	7.36899	32	*(27)*	*(27)*	*(7)*
WSe_2	114.0	2.80	160	/	0.13055	/	43	*(28)*	/	*(12)*
Bi_2O_2Se	105.8	2.65	~16	~1.76	0.00014713	0.00132	1.6	*(29)*	*(29)*	*(2)*
$FePS_3$	36.6	2.74	236	/	0.13202	/	1.35	*(30)*	/	*(3)*
$PtSe_2$	117.7	2.85	211.2	2.34	0.31632	1.72048	40.4	*(31)*	*(31)*	*(11)*
Black phosphorus	31.0	2.73	500	/	1.0598	/	20	*(32)*	/	*(13)*
Ti_2CO_2	27.9	2.17	356	~1.72	0.27315	15.25082	40.58*	*(33)*	*(33)*	*(14)*
$MoSi_2N_4$	29.7	2.24	436.45	0.77	0.55269	140.2239	173.03	*This work*		

Notes:

1. The value of κ of Ti_2CO_2 was derived from theoretical calculation result, while those of other materials were measured based on the optothermal Raman technique.
2. The values of δ^3 of $MoSi_2N_4$ and Ti_2CO_2 were obtained based on the first-principles calculations, while those of other materials were obtained from website of Materials Project database (https://nextgen.materialsproject.org).
3. Black phosphorus is an anisotropic material with reported thermal conductivity of ~20 $W \cdot m^{-1} \cdot K^{-1}$ (Zigzag direction) and ~10 $W \cdot m^{-1} \cdot K^{-1}$ (Armchair direction) for few-layer samples[13]. Monolayer black phosphorus has poor stability, so it is difficult to measure its thermal conductivity. Here we chose ~20 $W \cdot m^{-1} \cdot K^{-1}$ as the thermal conductivity of black phosphorus for comparison with other 2D semiconductors.

Supplementary Notes

Supplementary Note 1 | COMSOL simulations of temperature distribution profiles of monolayer $MoSi_2N_4$ suspended on holes.

We simulated the laser heating (schematic shown in Fig. 3a) using a finite element thermal simulation with COMSOL Multiphysics to obtain the temperature distribution profile of monolayer $MoSi_2N_4$ suspended on holes. The Fourier heat diffusion equations were solved with cylindrical coordinates (2D axisymmetric configuration) and a Gaussian-shaped beam heat source. The temperature increase of a suspended monolayer $MoSi_2N_4$ under laser excitation is directly related to its thermal conductivity, assuming that the absorbed heat transfers radially through a small cross-sectional area of the flake from the center to the edge.

As reported in previous work[11,34], the diffusion of heat in the suspended region can be expressed as the following equation:

$$\kappa \frac{1}{r}\frac{d}{dr}\left[r\frac{dT_1(r)}{dr}\right] + q(r) = 0 \qquad \text{for } r<R \tag{S1}$$

Where κ is the in-plane thermal conductivity of suspended monolayer $MoSi_2N_4$, $T_1(r)$ is the temperature distribution profile inside the hole (radius R), $q(r)$ is the volumetric optical heating and defined as Eq. (S2)

$$q(r) = \frac{I\alpha}{t} exp(-\frac{r^2}{r_0^2}) \tag{S2}$$

where $I = P/(\pi r_0^2)$ is the peak laser power per unit area at the center of beam spot, a is the optical absorption coefficient of monolayer $MoSi_2N_4$ at 532 nm, t is the thickness of monolayer $MoSi_2N_4$ flake, and r_0 = 0.59 μm is the laser beam radius used in our experimental system.

While outside the hole, the heat transports not only along the flake, but also into the Au/SiO_2/Si substrate, so its heat dissipation can be described as the following equation:

$$\kappa' \frac{1}{r}\frac{d}{dr}\left[r\frac{dT_2(r)}{dr}\right] - \frac{G}{t}[(T_2(r) - T_a)] = 0 \qquad \text{for } r>R \qquad \text{(S3)}$$

where κ' is the in-plane thermal conductivity of the supported monolayer $MoSi_2N_4$, $T_2(r)$ is the temperature distribution profile outside the hole, G is the interfacial thermal conductance between monolayer $MoSi_2N_4$ and Au/SiO_2/Si substrate, and T_a is the ambient temperature (300 K).

Consequently, the spatial distribution profiles of temperature inside the hole [$T_1(r)$] and outside the hole [$T_2(r)$] could be obtained by solving Eqs. (S1) and (S3), and the results are

$$T_1(r) = c_1 - 2c_2\ln(r) + c_3Ei(1, \left(\frac{r}{r_0}\right)^2) \qquad \text{for } r<R \qquad \text{(S4)}$$

$$T_2(r) = c_4K_0\left[0, r\sqrt{\frac{G}{\kappa' t}}\right] + T_a \qquad \text{for } r>R \qquad \text{(S5)}$$

where c_i are constants to be determined by boundary conditions, K_0 is the zero-order modified Bessel function of the second kind, and Ei is the exponential integral.

Taking into account the suitable boundary conditions ($r = R$ and $r \rightarrow \infty$):

$$T_1(R) = T_2(R) \qquad \text{(S6)}$$

$$T_2(r) = T_a \ (\mathrm{r} \rightarrow \infty) \qquad \text{(S7)}$$

the temperature profile for both inside the hole [$T_1(r)$] and outside the hole [$T_2(r)$] under different laser powers can be expressed with three unknown parameters κ, κ' and G. For κ', a reasonable assumption $\kappa' = \kappa$ was made, which has been adopted in the reported work[11,34]. Therefore, in our COMSOL simulation process, we input 173 W·m⁻

$^{1}\cdot K^{-1}$ as the values of κ and κ'. In addition, in the COMSOL simulation process, we need to input the value of out-of-plane thermal conductivity $\kappa_{\perp}$.

Next, we changed the assumed conditions for G and $\kappa_{\perp}$ in the COMSOL simulations, and the obtained temperature curves (Supplementary Fig. 6) are basically coincident at the same hole size and excitation power. This result indicates that the interface does not have an influence on the temperature distribution, which is consistent with those reported in previous work[11]. Thus, we chose a representative interface thermal conductivity (G) value of 100 $MW\cdot m^{-2}\cdot K^{-1}$ and $\kappa_{\perp}$ of 0.173 $W\cdot m^{-1}\cdot K^{-1}$. In fact, under the present experimental conditions, $\kappa_{\perp}$ of monolayer 2D materials cannot be directly measured. Previous work[35] has experimentally confirmed that the $\kappa_{\perp}$ values of multilayer MoS_2 (57 ± 3 $mW\cdot m^{-1}\cdot K^{-1}$) and WS_2 (41 ± 3$mW\cdot m^{-1}\cdot K^{-1}$)) are about 1/900 of their respective in-plane thermal conductivity. Therefore, here we assumed that the value of $\kappa_{\perp}$ is three orders of magnitude smaller than that of κ for monolayer $MoSi_2N_4$.

In our experiments, the maximum incident laser power was 3 mW, and the laser spot size was 1.18 μm. Under such experimental conditions, the temperature of monolayer $MoSi_2N_4$ near the hole edge for different hole size was simulated (Supplementary Fig. 7). It is worth noting that the temperature near the edge of the 6-μm-diametered hole reached 300.06 K (ambient temperature) (Supplementary Fig. 8), indicating that 6-μm-diametered hole is large enough to ensure the accuracy of thermal conductivity extraction[11].

In summary, the COMSOL simulation results imply that holes with a diameter of 6 μm are large enough for heat flow transfer from the center to the edge of monolayer $MoSi_2N_4$.

Supplementary Note 2 | Statistical errors evaluation of extracting thermal conductivity.

There are two kinds of sources for the statistical errors in our experimental tests, including the error of Voigt fitting in precise identification of the Raman peak position and the error of linear fitting of extracting first-order temperature and power coefficients. According to the Error Propagation Formula in Statistics, as $\varepsilon(x_1)$ and $\varepsilon(x_2)$ are the errors of x_1 and x_2, the statistical error of x_1/x_2 should be:

$$\varepsilon(x_1/x_2) \approx \frac{x_1 \cdot \varepsilon(x_2) - x_2 \cdot \varepsilon(x_1)}{x_2^2} \qquad (x_2 \neq 0) \tag{S8}$$

Taking the extracted data in our manuscript for example, the first-order temperature coefficient χ and laser power dependent coefficient $\delta\omega/\delta P$ are -0.01169 ± 0.00069 cm^{-1}/K and -0.55471 ± 0.01978 cm^{-1}/mW, respectively, thus $\chi/\left(\frac{\delta\omega}{\delta P}\right)$ should be 0.021074 ± 0.000492 $mW{\cdot}K^{-1}$. By substituting this value into Eq. (3) in the manuscript, the thermal conductivity of monolayer $MoSi_2N_4$ was calculated as 173.03 ± 4.04 $W{\cdot}m^{-1}{\cdot}K^{-1}$.

Supplementary Note 3 | Theoretical analyses on the influence of the position and density of N-vacancy on the thermal conductivity.

Based on Klemens P. G.'s perturbation theory[36], we revealed the influences of the position and density of N-vacancy on the thermal conductivity of monolayer $MoSi_2N_4$. The Matthiessen's rule was used to combine the different phonon scattering mechanisms, and therefore

$$\tau^{-1} = \tau_U^{-1} + \tau_B^{-1} + \tau_V^{-1} + \tau_A^{-1} \tag{S9}$$

Where τ^{-1} is the combined phonon scattering rate, τ_U^{-1} is the Umklapp phonon-phonon scattering rate, τ_B^{-1} is the phonon-boundary scattering rate, τ_V^{-1} is the phonon-vacancy scattering rate, and τ_A^{-1} is the phonon scattering rate caused by the change of force constant. For the suspended single crystal samples, we mainly focused on τ_U^{-1}, τ_V^{-1} and τ_A^{-1}.

Slack G. A. and Galginaitis S. suggested the following form for the Umklapp phonon–phonon scattering rate[37]:

$$\tau_U^{-1} = p\omega^2 \left(\frac{T}{\theta}\right) \exp\left(-\frac{\theta}{3T}\right) = C_U\omega^2 \tag{S10}$$

Where p is a constant, ω, T and θ are the circular frequency, temperature and Debye temperature, respectively. And C_U is the aggregation of other parameters except for ω.

Perturbation theory of Klemens P. G. described the phonon-vacancy scattering rate as[38]:

$$\tau_V^{-1} = x\left(\frac{\Delta M}{M}\right)^2 \frac{\pi}{2}\frac{\omega^2 g(\omega)}{G_N} \tag{S11}$$

Where x is the density of vacancies, $\frac{\Delta M}{M}$ is the effective mass, which is equal to $-\frac{M_a}{M} - 2$, M_a and M are the mass of vacancy atom and the average mass per atom,

respectively, $g(\omega)$ is the phonon density of states, and G_N is the number of atoms in the crystal.

Based on the bond-order theory for phonon-vacancy scattering[39-42], the scattering rate of phonons caused by the change of force constant was derived as:

$$\tau_A^{-1} = 4\pi zx\left\{\left[\frac{1+\exp[(12-z)/8z]}{1+\exp[(13-z)/(8z-8)]}\right]^{-(m+2)} - 1\right\}^2 \frac{\omega^2 g(\omega)}{G_N} \tag{S12}$$

Where z is the effective coordination number, m is a parameter that represents the nature of bond, for compounds, m is around 4.

In monolayer $MoSi_2N_4$, the nitrogen atoms have two different lattice sites, and the inner N_1 atom and the surface N_2 atom have effective coordination numbers of 4 and 3, respectively. Thus, N_1 and N_2 vacancies have the same τ_V^{-1} but different τ_A^{-1}. Based on Eq. (S11) and (S12), τ_A^{-1} is 1.55 and 9.36 times larger than τ_V^{-1} for N_1 and N_2 vacancies, respectively. Therefore, the scattering of phonons by N_2 vacancy is stronger than that of N_1 vacancy, which indicates that the existence of N_2 vacancy has a greater negative effect on the thermal conductivity of $MoSi_2N_4$.

In order to further demonstrate the influence of N-vacancy type and concentration on the phonon scattering and thermal conductivity, the parameters obtained from first-principles calculations at 300 K were extracted. Subsequently, we plotted the phonon scattering rate as a function of phonon frequency (Supplementary Fig. 15a) with a N-vacancy density of 0.01%. For the low frequency phonon scattering, the Umklapp phonon-phonon scattering is dominant. The phonon scattering by the change of force constant caused by N_2 vacancy plays a major role in the high frequency phonon scattering, while the N_1 vacancy has relatively little influence on the phonon scattering.

Additionally, we plotted the normalized thermal conductivity of $MoSi_2N_4$ as a function of N-vacancy density (Supplementary Fig. 15b). As the density of N_1 and N_2 vacancy increased, the normalized thermal conductivity decreased rapidly first and then decreased slowly. It is worth noting that N_2 vacancy caused a more pronounced decrease in thermal conductivity compared with N_1 vacancy.

Supplementary References

1. Yang, F. et al. Thermal transport and energy dissipation in two-dimensional Bi_2O_2Se. *Appl. Phys. Lett.* **115**, 193103 (2019).
2. Hossain, M. T. & Giri, P. K. Temperature-dependent Raman studies and thermal conductivity of direct CVD grown non-van der Waals layered Bi_2O_2Se. *J. Appl. Phys.* **129**, 175102 (2021).
3. Kargar, F. et al. Phonon and thermal properties of quasi-two-dimensional $FePS_3$ and $MnPS_3$ antiferromagnetic semiconductors. *ACS Nano* **14**, 2424-2435 (2020).
4. Su, Y. et al. Highly in-plane anisotropy of thermal transport in suspended ternary chalcogenide Ta_2NiS_5. *Nano Res.* **15**, 6601-6606 (2022).
5. Yan, Z. et al. Phonon and thermal properties of exfoliated $TaSe_2$ thin films. *J. Appl. Phys.* **114**, 204301 (2013).
6. Botcha, V. D. et al. High-K substrate effect on thermal properties of 2D InSe few layer. *J. Alloys Compd.* **735**, 594-599 (2018).
7. Peimyoo, N. et al. Thermal conductivity determination of suspended mono- and bilayer WS_2 by Raman spectroscopy. *Nano Res.* **8**, 1210-1221 (2015).
8. Yu, Y., Minhaj, T., Huang, L., Yu, Y. & Cao, L. In-plane and interfacial thermal conduction of two-dimensional transition-metal dichalcogenides. *Phys. Rev. Appl.* **13**, 034059 (2020).
9. Li, X. et al. Isotope-engineering the thermal conductivity of two-dimensional MoS_2. *ACS Nano* **13**, 2481-2489 (2019).

10. Zhang, X. et al. Measurement of lateral and interfacial thermal conductivity of single- and bilayer MoS_2 and $MoSe_2$ using refined optothermal Raman technique. *ACS Appl. Mater. Interfaces* **7**, 25923-25929 (2015).
11. Yin, S. et al. Thermal conductivity of few-layer PtS_2 and $PtSe_2$ obtained from optothermal Raman spectroscopy. *J. Phys. Chem. C* **125**, 16129-16135 (2021).
12. Wang, Y. et al. Thermal conductivities and interfacial thermal conductance of 2D WSe_2. In *IEEE 15th International Conference on Nano/Micro Engineered and Molecular System (NEMS)* 575-579 (IEEE, 2020).
13. Luo, Z. et al. Anisotropic in-plane thermal conductivity observed in few-layer black phosphorus. *Nat. Commun.* **6**, 8572 (2015).
14. Sarikurt, S., Cakir, D., Keceli, M. & Sevik, C. The influence of surface functionalization on thermal transport and thermoelectric properties of MXene monolayers. *Nanoscale* **10**, 8859-8868 (2018).
15. Sagar, R. U. R. et al. Transfer-free growth of Bi_2O_2Se on silicon dioxide via chemical vapor deposition. *ACS Appl. Electron. Mater.* **2**, 2123-2131 (2020).
16. Li, Y. et al. Elastic properties and intrinsic strength of two-dimensional InSe flakes. *Nanotechnology* **30**, 335703 (2019).
17. Yang, Y. et al. Brittle fracture of 2D $MoSe_2$. *Adv. Mater.* **29**, 1604201 (2017).
18. Falin, A. et al. Mechanical properties of atomically thin tungsten dichalcogenides: WS_2, WSe_2, and WTe_2. *ACS Nano* **15**, 2600-2610 (2021).
19. Bertolazzi, S., Brivio, J. & Kis, A. Stretching and breaking of ultrathin MoS_2. *ACS Nano* **5**, 9703-9709 (2011).
20. Liu, K. et al. Elastic properties of chemical-vapor-deposited monolayer MoS_2, WS_2, and their bilayer heterostructures. *Nano Lett.* **14**, 5097-5103 (2014).
21. Das, S. et al. Raman and first-principles study of the pressure-induced Mott-insulator to metal transition in bulk $FePS_3$. *J. Phys. Chem. Solids* **164**, 110607 (2022).
22. Nguyen, D.-T. Elastic mechanical properties of transition metal dichalcogenides monolayer using atomic finite element method. In *2021 AUN/SEED-Net Joint Regional Conference in Transportation, Energy, and Mechanical Manufacturing*

Engineering (RCTEMME 2021) 687-700 (2022).

23. Chang, X., Ji, Y., Jia, M. & Li, H. Molecular dynamics simulations for mechanical properties of the monolayer PtS_2 with line defect. *Comput. Mater. Sci.* **214**, 111734 (2022).
24. Khaledialidusti, R., Anasori, B. & Barnoush, A. Temperature-dependent mechanical properties of $Ti_{n+1}C_nO_2$ (n = 1, 2) MXene monolayers: a first-principles study. *Phys. Chem. Chem. Phys.* **22**, 3414-3424 (2020).
25. Jiang, J. W. & Park, H. S. Mechanical properties of single-layer black phosphorus. *J. Phys. D: Appl. Phys.* **47**, 385304 (2014).
26. Hong, Y.-L. et al. Chemical vapor deposition of layered two-dimensional $MoSi_2N_4$ materials. *Science* **369**, 670-674 (2020).
27. Peng, B. et al. Thermal conductivity of monolayer MoS_2, $MoSe_2$, and WS_2: interplay of mass effect, interatomic bonding and anharmonicity. *RSC Adv.* **6**, 5767-5773 (2016).
28. Mathew, S. et al. Temperature dependent structural evolution of WSe_2: a synchrotron X-ray diffraction study. *Condens. Matter* **5**, 76 (2020).
29. Wang, N. et al. Optimizing the thermoelectric transport properties of Bi_2O_2Se monolayer via biaxial strain. *Phys. Chem. Chem. Phys.* **21**, 15097-15105 (2019).
30. Takano, Y. et al. Magnetic properties and specific heat of MPS_3 (M=Mn, Fe, Zn). *J. Magn. Magn. Mat.* **272-276**, E593–E595 (2004).
31. Lei, J.-Q., Liu, K., Huang, S. & Zhou, X.-L. The comparative study on bulk-$PtSe_2$ and 2D 1-Layer-$PtSe_2$ under high pressure via first-principle calculations. *Theor. Chem. Acc.* **136**, 97 (2017).
32. Jain, A. & McGaughey, A. J. Strongly anisotropic in-plane thermal transport in single-layer black phosphorene. *Sci. Rep.* **5**, 8501 (2015).
33. Guo, Z., Miao, N., Zhou, J., Pan, Y. & Sun, Z. Coincident modulation of lattice and electron thermal transport performance in MXenes via surface functionalization. *Phys. Chem. Chem. Phys.* **20**, 19689-19697 (2018).
34. Yan, R. *et al.* Thermal conductivity of monolayer molybdenum disulfide obtained

from temperature-dependent Raman spectroscopy. *ACS Nano* **8**, 986-993 (2014).

35. Kim, S. E. *et al.* Extremely anisotropic van der Waals thermal conductors. *Nature* **597**, 660-665 (2021).

36 Ratsifaritana, C. A. & Klemens, P. G. Scattering of phonons by vacancies. *Int. J. Thermophys.* **8**, 737-750 (1987).

37. Slack, G. A. & Galginaitis, S. Thermal conductivity and phonon scattering by magnetic impurities in CdTe. *Phys. Rev.* **133**, A253-A268 (1964).

38. Klemens, P. G. & Pedraza, D. F. Thermal-conductivity of graphite in the basal-plane. *Carbon* **32**, 735-741 (1994).

39. Sun, C. Q. *et al.* Bond-order-bond-length-bond-strength (bond-OLS) correlation mechanism for the shape-and-size dependence of a nanosolid. *J. Phys.: Condens. Matter* **14**, 7781-7795 (2002).

40. Sun, C. Q., Li, C. M., Bai, H. L. & Jiang, E. Y. Melting point oscillation of a solid over the whole range of sizes. *Nanotechnology* **16**, 1290-1293 (2005).

41. Sun, C. Q. Size dependence of nanostructures: Impact of bond order deficiency. *Prog. Solid State Chem.* **35**, 1-159 (2007).

42. Xie, G. F. *et al.* A bond-order theory on the phonon scattering by vacancies in two-dimensional materials. *Sci. Rep.* **4**, 5085 (2014).
