# Supplementary material for: Unusually high thermal conductivity in suspended monolayer MoSi2N4
Source: Nat Commun. 2024 Jun 6;15:4832. doi: 10.1038/s41467-024-48888-9 (PMC11156898; doi:10.1038/s41467-024-48888-9)
Supplement: Supplementary file 3 — Lasing Reporting Summary [file 41467_2024_48888_MOESM3_ESM.pdf]

Corresponding author(s): Wencai Ren

Lasing Reporting Summary

Nature Research wishes to improve the reproducibility of the work that we publish. This form is intended for publication with all accepted papers reporting claims of lasing and provides structure for consistency and transparency in reporting. Some list items might not apply to an individual manuscript, but all fields must be completed for clarity.

For further information on Nature Research policies, including our data availability policy, see Authors & Referees.

• Experimental design

Please check: are the following details reported in the manuscript?

1. Threshold

Plots of device output power versus pump power over a wide range of values indicating a clear threshold

☐ Yes
☒ No

The maximum output power is about 23 mW, which is large enough for the maximum power of 3mW used in our optothermal Raman measurements. Besides, we are not clear about the specific pump power of the Raman device. We just need to make sure that the device can meet the maximum power we use.

2. Linewidth narrowing

Plots of spectral power density for the emission at pump powers below, around, and above the lasing threshold, indicating a clear linewidth narrowing at threshold

☐ Yes
☒ No

We are not professional in Raman devices. The engineers of Witec Raman company are responsible for installation, debugging and training to ensure the normal and valid operation. Under the right working conditions, we make sure that our experimental testing and results are reliable. The "Linewidth narrowing" are not relevant in our work.

Resolution of the spectrometer used to make spectral measurements

☐ Yes
☒ No

We are not professional in Raman devices. The engineers of Witec Raman company are responsible for installation, debugging and training to ensure the normal and valid operation. Under the right working conditions, we make sure that our experimental testing and results are reliable. The "Resolution" is not the subject of our study.

3. Coherent emission

Measurements of the coherence and/or polarization of the emission

☐ Yes
☒ No

We are not professional in Raman devices. The engineers of Witec Raman company are responsible for installation, debugging and training to ensure the normal and valid operation. We make sure that our experimental testing and results are reliable.

4. Beam spatial profile

Image and/or measurement of the spatial shape and profile of the emission, showing a well-defined beam above threshold

☒ Yes
☐ No

This information can be found in the part of "Thermal conductivity measurements " of "Methods" section in the manuscript. A diffraction-limit spot size of the 532 nm laser is about 1.18 µm with a long-working-distance 50x objective (NA = 0.55), according to the engineers of Witec Raman company .

5. Operating conditions

Description of the laser and pumping conditions
Continuous-wave, pulsed, temperature of operation

☒ Yes
☐ No

This information can be found in the part of "Thermal conductivity measurements " of "Methods" section in the manuscript.

Threshold values provided as density values (e.g. W cm^{-2} or J cm^{-2}) taking into account the area of the device

☐ Yes
☒ No

The maximum output power is about 23 mW, which is large enough for the maximum power of 3mW used in our optothermal Raman measurements. Besides, we are not clear about the specific pump power of the Raman device. We just need to make sure that the device can meet the maximum power we use.

6. Alternative explanations

Reasoning as to why alternative explanations have been ruled out as responsible for the emission characteristics
e.g. amplified spontaneous, directional scattering; modification of fluorescence spectrum by the cavity

☐ Yes
☒ No

We are not professional in Raman devices. The engineers of Witec Raman company are responsible for installation, debugging and training to ensure the normal and valid operation. Under the right working conditions, we make sure that our experimental testing and results are reliable. The "Alternative explanations" are not relevant in our work.

7. Theoretical analysis

Theoretical analysis that ensures that the experimental values measured are realistic and reasonable
e.g. laser threshold, linewidth, cavity gain-loss, efficiency

☐ Yes
☒ No

We are not professional in Raman devices. The engineers of Witec Raman company are responsible for installation, debugging and training to ensure the normal and valid operation. We ensure that the experimental values measured in our work are realistic and reasonable.

8. Statistics

Number of devices fabricated and tested	☐ Yes ☒ No	Raman spectra were carried out on WITec alpha300R confocal Raman microscopy (Oxford WITec).
Statistical analysis of the device performance and lifetime (time to failure)	☐ Yes ☒ No	We are not professional in Raman devices. The engineers of Witec Raman company are responsible for installation, debugging and training to ensure the normal and valid operation. Under the right working conditions, we make sure that our experimental testing and results are reliable. The description of "Statistical analysis of the device performance and lifetime (time to failure)" is the responsibility of Witec Raman company and its development engineers.
